# Lung development in the late preterm

**DOI:** 10.1186/1824-7288-40-S2-A24

**Published:** 2014-10-09

**Authors:** Maurizio Gente, Paola Papoff, Stefano Luciani, Rosanna Grossi, Elena Caresta, Corrado Moretti

**Affiliations:** 1 Department of Paediatrics, Neonatal Emergency Transport Service, Sapienza University of Rome, Rome, Italy; 2 Department of Paediatrics, Paediatric Emergency and Intensive Care, Sapienza University of Rome, Rome, Italy

## 

An increasing incidence of moderate-to-late prematurity is observed worldwide (6-7% of all births). Moderate-to-late prematurity is a cause of important mortality and morbidity, even when it is just a few weeks before term gestation [1]. Respiratory issues related to moderate prematurity include delayed neonatal transition to air breathing, respiratory distress resulting from delayed fluid clearance, surfactant deficiency, and pulmonary hypertension. There is increasing evidence to support the hypothesis that preterm delivery, even in the absence of any neonatal respiratory disease, may have adverse effects on subsequent lung growth and development, and that these alterations may persist during the early years of life. Premature birth interrupts normal in utero lung development and results in an early transition from the hypoxic intrauterine environment to a comparatively hyperoxic atmospheric environment [2]. Alveolar walls may be thicker, impairing optimal gas exchange. Colin et al. proposed that preterm birth leads to decreased parenchyma elasticity and subsequent airway tethering, a mechanism by which airway wall compliance keeps surrounding alveoli well opened [3]. The long-term significance of reduced airway function early in life has been emphasized in a longitudinal study involving a large group of non-selected infants who had participated in the Tucson Children’s Respiratory study [4]. In this study Stern et al. showed that infants whose pulmonary function was in the lowest quartile also had pulmonary function in the lowest quartile through the years of follow-up until early adulthood. These findings in a normal unselected population, suggest that the level of pulmonary function in early life tracks and changes little with growth. Several authors suggest that deficits in lung function during early life, especially if associated with lower respiratory illnesses, increase the risk of chronic obstructive pulmonary disease in late adult life [5-7]. Of particular importance in this context may be the role played by RSV, which affects most children during their first year of life. The risk of life-threatening RSV infection appears relevant up to a post-conceptional age of 44 weeks. Stein et al. reported that RSV lower respiratory tract illness during the first 3 years of life in a healthy birth cohort was associated with recurrent wheeze up to age 11 [8].

In conclusion altered lung development is a characteristic feature of the late preterm infants and its impact on neonatal and postnatal morbidity needs to be considered.
